# Efficient Analytical Pretreatment of Cr(VI) in Ethylene Wastewater by Grafting g-C_3_N_4_ Material Based on Coupling Agent-Modified Basalt Matrix (Basalt–MTES/g-C_3_N_4_)

**DOI:** 10.3390/molecules30112477

**Published:** 2025-06-05

**Authors:** Zheng Wang, Mingchang Jia, Yi Ren, Hongmin Ren, Shuhao Liang, Jiaru Sun, Siqi Hao, Jinchuan Li, He Li

**Affiliations:** Department of Chemical Engineering, Hebei Petroleum University of Technology, Chengde 067000, China; 18789286658@163.com (Z.W.); 15532769658@163.com (Y.R.); 15831463175@163.com (H.R.); 15032363962@163.com (S.L.); 17320912605@163.com (J.S.); haosqcdpc123@yeah.net (S.H.); 17733750705@163.com (J.L.); 18832423881@163.com (H.L.)

**Keywords:** basalt–MTES/g-C_3_N_4_, Cr(VI), ethylene wastewater, analytical pretreatment, ion exchange

## Abstract

This study presents a novel Basalt-based grafted graphitic carbon nitride composite (Basalt–MTES/g-C_3_N_4_) for the efficient pretreatment of Cr(VI) in ethylene wastewater. The composite was synthesized by the acid purification of natural Basalt, surface modification with hydroxymethyl triethoxysilane (MTES), and the subsequent grafting of g-C_3_N_4_. Characterization confirmed the uniform distribution of nano-sized g-C_3_N_4_ particles on a Basalt surface with intact chemical bonding, where 82.63% of melamine participated in g-C_3_N_4_ crystallization. The material exhibited a high specific surface area (403.55 m^2^/g) and mesoporous structure (34.29 nm). Acidic conditions promoted the protonation of amino groups in g-C_3_N_4_, significantly enhancing Cr(VI) adsorption via ion exchange. Adsorption kinetics followed the pseudo-second-order model, while isotherm data fitted the Langmuir monolayer adsorption mechanism. The composite achieved 97% Cr(VI) recovery through chromatographic extraction and retained 96.87% removal efficiency after five regeneration cycles. This work demonstrates a cost-effective, recyclable green pretreatment material for high-sensitivity Cr(VI) monitoring in ethylene industry wastewater, offering dual benefits in environmental remediation and regulatory compliance. The design synergizes natural Basalt’s stability with g-C_3_N_4_’s adsorption affinity, showing practical potential for sustainable wastewater treatment technologies.

## 1. Introduction

As the core product of the modern petrochemical industry, ethylene’s production scale directly reflects the industrialization level of a country. The global ethylene production capacity has been continuously increasing in recent years, reaching 210 million tons in 2023, and is widely used in plastics, synthetic rubber, fibers, and other fields [1]. However, at the same time, the environmental monitoring issues involved in the ethylene refining process have to be given increasing attention. According to statistics, as much as 18.2 billion tons of wastewater is generated globally each year from the refining of ethylene (2017) [2]. The industrial wastewater produced during the ethylene refining process is complex in composition, containing a large amount of refractory organic matter and heavy metal ions, posing a serious threat to the ecosystem and human health [3]. Although various countries have set strict standards for the discharge of ethylene wastewater (for instance, China’s ’Pollutant Discharge Standard for Petrochemical Industry’ stipulates that the COD limit is 60 mg·L^−1^), there are still problems, such as the strong concealment of pollutants and insufficient detection sensitivity in actual monitoring [4]. In particular, the trace analysis of highly toxic substances like Cr(VI) has become difficult in environmental monitoring [5].

In the monitoring of heavy metals in ethylene wastewater, Cr(VI) is listed as a priority pollutant to be controlled due to its strong oxidizing property, carcinogenicity, and bioaccumulation [6]. Current standards (such as the EU’s “Industrial Emission Directive”) require that the emission concentration of Cr(VI) shall not exceed 0.01 mg·L^−1^, which is much lower than the limits of metals such as iron (2.0 mg·L^−1^) and zinc (1.0 mg·L^−1^) [7]. However, Cr(VI) in ethylene wastewater often coexists with Fe^3+^, Cu^2+^, etc., and is disturbed by factors such as pH and organic matter [8]. Traditional spectrophotometric methods (such as the diphenylcarbazide method) are prone to false positivity or insufficient sensitivity [9]. Therefore, developing efficient enrichment and selective separation techniques has become the key to achieving the precise analysis of trace Cr(VI).

At present, the analytical pretreatment of Cr(VI) in ethylene production enterprises (such as the Sinopec Yanshan Petrochemical Company) mainly relies on the evaporation concentration method (simple principle but cumbersome operation), the ion exchange method (difficult resin regeneration), and the membrane enrichment method (severe membrane fouling) [10,11]. Although the extraction chromatography method has excellent selectivity, it requires the synthesis of specific functionalized eluent resins (such as those containing amino chelating groups), which makes the process complex and costly [12]. In recent years, graphite carbon nitride (g-C_3_N_4_) has shown a specific treatment capacity for Cr(VI) due to its layered structure rich in Lewis bases. Most applications of g-C_3_N_4_ in Cr(VI) treatment mainly focus on the reduction in Cr(VI) under photocatalysis, and studies have shown that based on the possible ion exchange or coordination complexation between the g-C_3_N_4_ functional group and Cr_2_O_7_^2−^ or CrO_4_^2−^, it also has great potential in the adsorption and analytical pretreatment applications of Cr(VI) [13,14]. However, its powder form is prone to agglomeration and difficult to recover, which limits its practical application. If it is immobilized on the surface of the porous matrix material, it can not only retain the adsorption activity but also improve the stability of the material. This idea provides new thoughts for the design and synthesis of new analytical pretreatment materials.

In recent studies, Basalt has become an ideal matrix for functional materials due to its natural porous structure, high mechanical strength, and surface-active groups [15]. Firstly, its natural porous structure (with pore diameters ranging from 20 to 80 nm) significantly enhances the adsorption capacity and rate, which is because it provides a high specific surface area (up to over 550 m^2^·g^−1^) and abundant mass transfer channels [16]. It is not uncommon for the matrix of porous structural materials to provide excellent reactive performance. For example, in the study of mesoporous carbon CMK-3 encapsulating nickel nanoparticles and homopoly (vinylsulfonic acid) as acid–metal bifunctional reduction amination catalysts by Kalbasi R. et al., the excellent pore structure played a significant promoting role in the catalytic effect [17]. Secondly, Basalt has excellent mechanical strength (tensile strength ≥ 3000 MPa) and thermal stability (temperature resistance > 600 °C) [18]. It can maintain structural integrity even under dynamic water flow or complex working conditions, avoiding secondary pollution caused by material breakage. The chemical inertness characteristic enables it to remain stable in the industrial wastewater environment, thereby extending the service life of the material. Furthermore, the abundant hydroxyl groups (with a -OH density of approximately 3.2/nm^2^) on the surface of Basalt can be directionally modified by silane coupling agents, providing an ideal interface for the loading of functional materials (such as g-C_3_N_4_) and achieving the precise regulation of adsorption performance [19]. In this study, Basalt material was used as the framework. The Basalt material was modified by the coupling agent hydroxymethyl triethoxysilane (MTES), and g-C_3_N_4_ was successfully grafted to construct a composite material (Basalt–MTES/g-C_3_N_4_) with both adsorption and separation functions for the pretreatment process of Cr(VI) analysis and measurement in ethylene wastewater. The surface conformation and synthesis mechanism of Basalt–MTES/g-C_3_N_4_ were analyzed by characterization methods such as SEM, FTIR, XRD, XPS, and BET. In terms of the application of synthetic materials, this study systematically evaluated the effects of Basalt–MTES/g-C_3_N_4_ on the acidity, isotherms, kinetics, adsorption heat, and regeneration performance of the Cr(VI) adsorption process, applied it to the analytical pretreatment of actual ethylene wastewater, and significantly improved the detection accuracy of the flame atomic absorption spectrophotometer. This research provides a design idea of new pretreatment materials for the high-sensitivity analysis of Cr(VI) in ethylene wastewater, which is of great practical significance for promoting the green production and environmental supervision technology progress in the ethylene industry.

**Figure 1:**
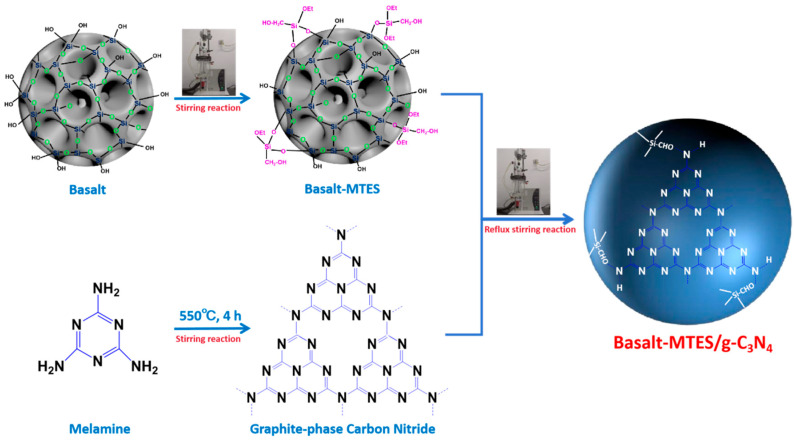
Synthesis process of Basalt–MTES/g-C_3_N_4_.

The synthesis flowchart of Basalt–MTES/g-C_3_N_4_ is shown in Figure 1.

## 2. Results and Discussion

### 2.1. Characterization of Basalt, Basalt–MTES, and Basalt–MTES/g-C_3_N_4_

**Figure 2:**
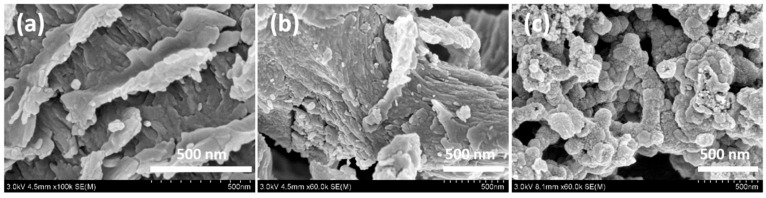
SEM images of Basalt (**a**), Basalt–MTES (**b**), and Basalt–MTES/g-C_3_N_4_ (**c**).

The SEM characterization of Basalt, Basalt–MTES, and Basalt–MTES/g-C_3_N_4_ is shown in Figure 2. For the Basalt (Figure 2a), since it has been thoroughly treated with HNO_3_, the soluble impurity components in the Basalt material are removed. Through this process, a large number of pores were formed on the surface and inside the material, and the specific surface area was greatly increased compared to before. After removing the impurities, the surface of the Basalt showed a lamellar porous structure. For Basalt–MTES (Figure 2b), because the grafting reaction of the coupling agent belongs to the small-molecule grafting reaction, the appearance of Basalt did not change significantly after the introduction of the coupling agent MTES. However, after further grafting g-C_3_N_4_, the surface of the material changed significantly. Since the formation process of carbon nitride belongs to the polymerization growth reaction, g-C_3_N_4_ will eventually be grafted on the surface of the matrix material in the form of a polymer, and the surface of the material will be covered. As shown in Figure 2c, the nano-sized granular material on the surface is a g-C_3_N_4_ polymer particle [20]. Furthermore, based on visual inspection, the diameter of the g-C_3_N_4_ particles is mostly less than 100 nm.

**Figure 3:**
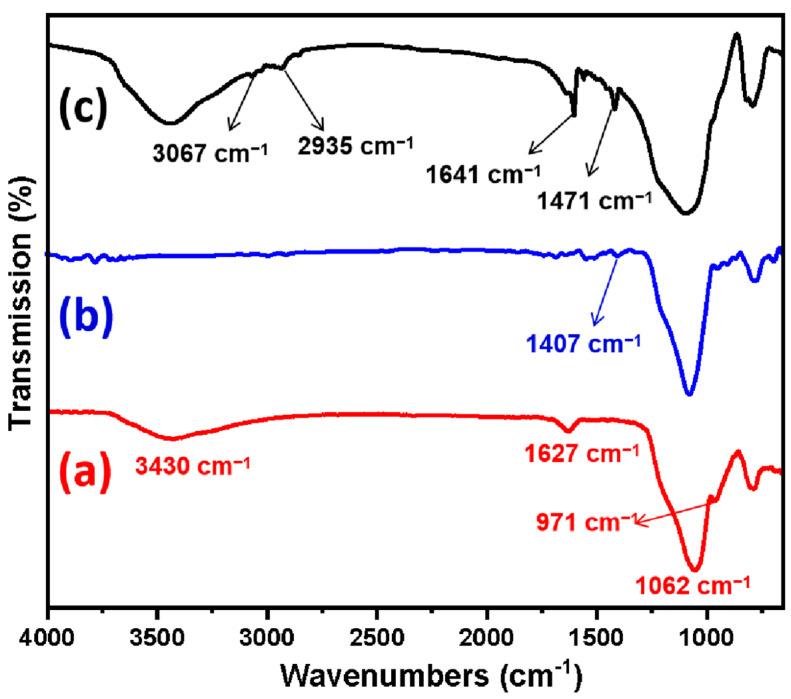
FTIR spectra of Basalt (**a**), Basalt–MTES (**b**), and Basalt–MTES/g-C_3_N_4_ (**c**).

The FTIR spectra of the Basalt, Basalt–MTES, and Basalt–MTES/g-C_3_N_4_ are shown in Figure 3. For the Basalt (Figure 3a), 1062 cm^−1^ is the stretching vibration peak of Si-O-Si, which is because Basalt is mainly composed of SiO_2_ (45–52%) and a variety of metal oxides (Al_2_O_3_, FeO, Fe_2_O_3_, CaO, MgO, etc.). After advanced treatment with HNO_3_, the metal oxides were dissolved, and the main framework of SiO_2_ was retained [21]. Overall, 971 cm^−1^ is the flexural vibration peak of -OH, 1627 cm^−1^ is the in-plane deformation vibration peak of -OH, and the broad peak around 3430 cm^−1^ is the stretching vibration peak of the double-base -OH and H_2_O [22,23]. This information indicates that the acid treatment process retained the SiO_2_ framework of Basalt and shows that after the acidification of HNO_3_, the surface of Basalt is rich in -OH.

For the Basalt–MTES (Figure 3b), the flexural vibration peak of -OH at 971 cm^−1^ and the in-plane deformation vibration peak of -OH at 1627 cm^−1^ were significantly weakened or disappeared, which indicates that the large amount of -OH on the surface of Basalt may react with the coupling agent MTES, and in this reaction, the -OCH_3_ in the coupling agent molecule condenses with the -OH on the surface of Basalt, and the two are chemically bonded together in the form of Si-O-Si [24]. The reaction is as follows:

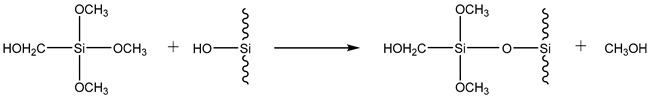



The wide peak at 3430 cm^−1^ disappears, indicating the disappearance of moisture on the surface of Basalt; that is, there may be a large number of organic hydrophobic groups (coupling agent molecules) on the surface of Basalt [25]. The weak peak at 1407 cm^−1^ is the in-plane bending vibration peak of C-H in -CH_3_ (due to the single-layer grafting of the coupling agent molecules, the peak intensity is relatively weak). The above information initially indicates that the coupling agent MTES has been grafted onto the surface of Basalt.

For the Basalt–MTES/g-C_3_N_4_ (Figure 3c), the characteristic peak at 1471 cm^−1^ is the C-C stretching vibration in the melamine ring outside, and the characteristic peak of 1641 cm^−1^ is the C-N stretching vibration in the melamine inside the ring [26]. In addition, 3067 cm^−1^ is the stretching vibration peak of C-H on -CH_2_-, and 2935 cm^−1^ is the stretching vibration peak of C-H in -CH_2_OH. This peak position information indicates that melamine has successfully grafted and polymerized on the material surface.

**Figure 4:**
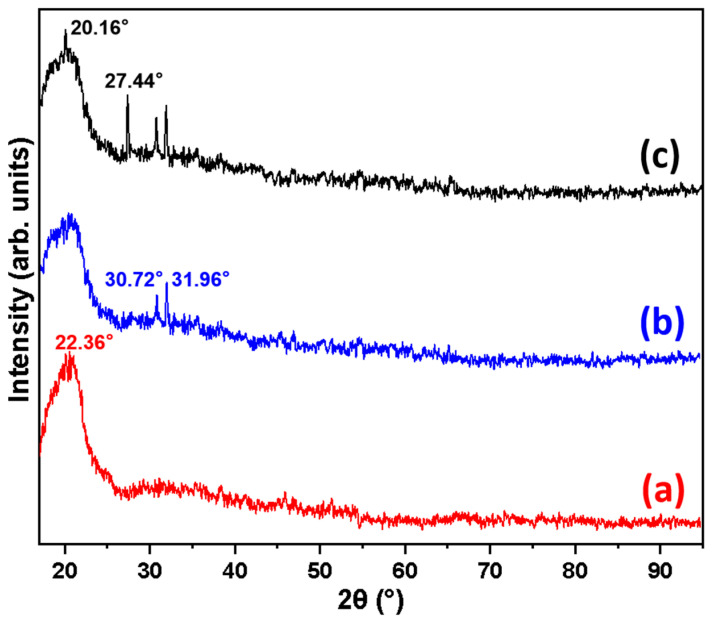
XRD pattern of Basalt (**a**), Basalt–MTES (**b**), and Basalt–MTES/g-C_3_N_4_ (**c**).

The XRD analysis results of the Basalt, Basalt–MTES, and Basalt–MTES/g-C_3_N_4_ are shown in Figure 4. For Basalt (Figure 4a), there is only one main characteristic peak of 22.36° in the spectrum, which is a typical feature peak of amorphous SiO_2_. This indicates that only SiO_2_ remains in the Basalt matrix substrate after acid treatment [27]. After grafting the coupling agent MTES, for the spectra of Basalt–MTES (Figure 4b), there are clearly organic compound diffraction peaks at 30.72° and 31.96°, which indicates that the coupling agent molecules are already present on the surface of the material [28]. For Basalt–MTES/g-C_3_N_4_ (Figure 4c), characteristic peaks appeared at 20.16° and 27.44°. The diffraction peak at 20.1° belongs to the microcrystalline structure of g-C_3_N_4_, and the diffraction peak at 27.4° corresponds to the (002) crystal plane, which belongs to the interlayer superposition of g-C_3_N_4_ [29]. This indicates that g-C_3_N_4_ and Basalt–MTES have successfully recombined. This analysis result is basically consistent with the FTIR result.

**Figure 5:**
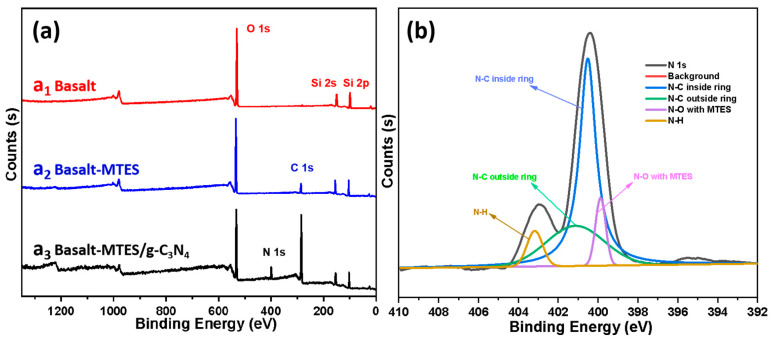
XPS spectra of Basalt, Basalt–MTES, and Basalt–MTES/g-C_3_N_4_ (**a**); the deconvolution of N 1 s spectra (**b**).

The XPS spectrogram of Basalt, Basalt–MTES, and Basalt–MTES/g-C_3_N_4_ is shown in Figure 5a, and the N 1s spectrum of Basalt–MTES/g-C_3_N_4_ is shown in Figure 5b. In Figure 5a, for Basalt (a_1_), there are only two elements: Si and O. Among them, the binding energy 103 eV is the Si 2p absorption peak, 153 eV is the Si 2s absorption peak, and 533 eV is the O 1s absorption peak, which indicates that the main framework component of the Basalt matrix after acid treatment is SiO_2_ [30]. Compared with Basalt, for the Basalt–MTES (a_2_) spectrogram, in addition to the Si and O absorption peaks, there is also the C 1s absorption peak, at 285 eV, indicating that the coupling agent molecules may already exist on the surface of Basalt [31]. For Basalt–MTES/g-C_3_N_4_ (a_3_), besides the above-mentioned peaks, the absorption peak of N 1s appears at 399 eV, which indicates that the melamine was successfully grafted to the surface of the Basalt [32].

In order to further explore the law of the grafting reaction of melamine on the surface of Basalt, we fitted the N 1s absorption peak of Basalt–MTES/g-C_3_N_4_. After the grafting reaction, the N in the material may have the following four connection modes, which are the N-C inside the melamine ring, the N-C outside the melamine ring, the N-O connected with the coupling agent, and the N-H not involved in the reaction. Four deconvoluted peaks centered at 400.5 eV, 401.2 eV, 399.9 eV, and 403.2 eV were assigned to the above four connection methods, respectively [33,34]. The N 1s spectra of Basalt–MTES/g-C_3_N_4_ are illustrated in Figure 5b. According to the fitting results, the peak area ratios of the four groups are 77.12%, 10.51%, 3.85%, and 8.52%, respectively. Among the melamine grafted on the surface of Basalt, the proportion of melamine involved in g-C_3_N_4_ crystal formation reached 82.63%.

**Figure 6:**
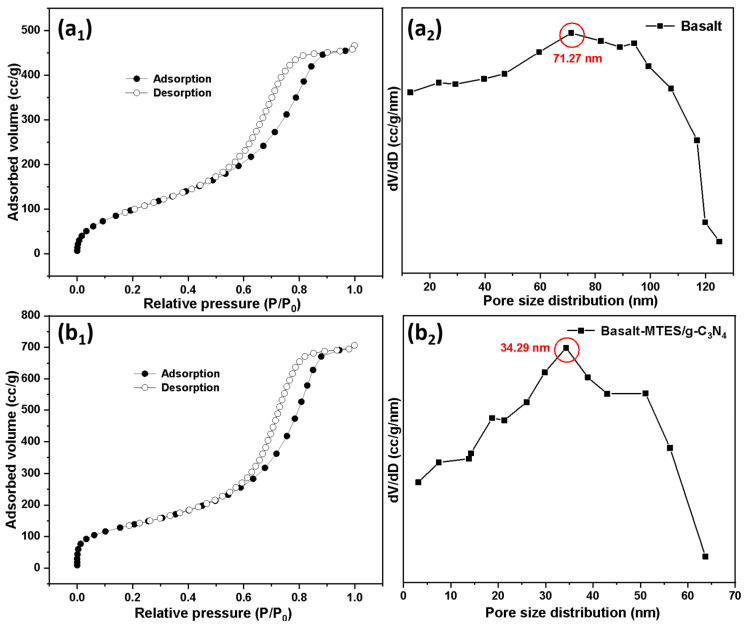
Nitrogen sorption isotherm and pore size distribution of Basalt (**a_1_**,**a_2_**) and Basalt–MTES/g-C_3_N_4_ (**b_1_**,**b_2_**).

Nitrogen sorption isotherm and pore size distribution of Basalt and Basalt–MTES/g-C_3_N_4_ are shown in Figure 6. For Basalt (Figure 6(a_1_,a_2_)), the specific surface area and maximum probability aperture calculated by the BET method and the BJH method are 563.25 m^2^·g^−1^ and 71.27 nm, respectively. After the grafting reaction (Figure 6(b_1_,b_2_)), the pore size and specific surface area decrease, as shown in Table 1. After the melamine grafting reaction, the material still has a large pore size and specific surface area (406.55 m^2^·g^−1^, 34.29 nm), which can fully ensure that the adsorbed substance can freely enter the pore interior.

### 2.2. Adsorption Experiment Results of Cr(VI) by Basalt–MTES/g-C_3_N_4_

**Figure 7:**
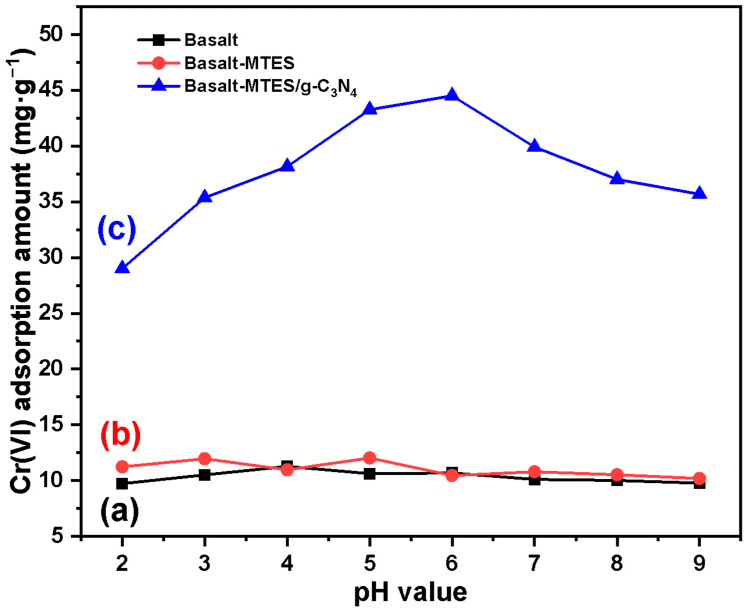
The influence of pH on the adsorption of Cr(VI) by Basalt (**a**), Basalt–MTES (**b**), and Basalt–MTES/g-C_3_N_4_ (**c**).

Figure 7 examines the influence of pH (2–9) on the adsorption of Cr(VI) by three materials: Basalt, Basalt–MTES, and Basalt–MTES/g-C_3_N_4_. It can be seen from the figure that under any pH conditions, the adsorption effect of Basalt–MTES/g-C_3_N_4_ on Cr(VI) is much higher than that of Basalt and Basalt–MTES, which indicates that the g-C_3_N_4_ group has a significant adsorption effect on Cr(VI) [35]. For the adsorption of Cr(VI) by Basalt–MTES/g-C_3_N_4_ (Figure 7c), with the increase in pH, the adsorption amount first increases and then decreases. The adsorption amount gradually increases within the pH range of 2 to 6, which is because under low pH (2–4) conditions, Cr(VI) mainly exists in the form of Cr_2_O_7_^2−^, and its strong oxidizing property causes a certain degree of damage to the organic functional groups in the material, so the adsorption effect is not ideal. And as the pH increases to 5–6, part of Cr_2_O_7_^2−^ is converted into CrO_4_^2−^. Meanwhile, the oxidizing power of CrO_4_^2−^ is limited when the acidity weakens, the damage to the functional groups of the material is reduced, and the adsorption effect is significantly improved. When pH > 6, Cr_2_O_7_^2−^ is converted in large quantities to CrO_4_^2−^; that is, the concentration of the adsorbate increases, which leads to a certain degree of decline in the adsorption effect within the limited adsorption sites [36]. As the pH continues to rise, the adsorption effect further declines, and the alkalinity of the solution increases. This is because the SiO_2_ in the Basalt matrix is corroded under alkaline conditions, resulting in a certain degree of damage to the material framework (pore structure) and affecting the adsorption effect. And as the alkalinity increases, the adsorption capacity decreases, indicating that the material is more damaged. Therefore, pH 5–6 is more conducive to the removal of Cr(VI) by Basalt–MTES/g-C_3_N_4_.

**Figure 8:**
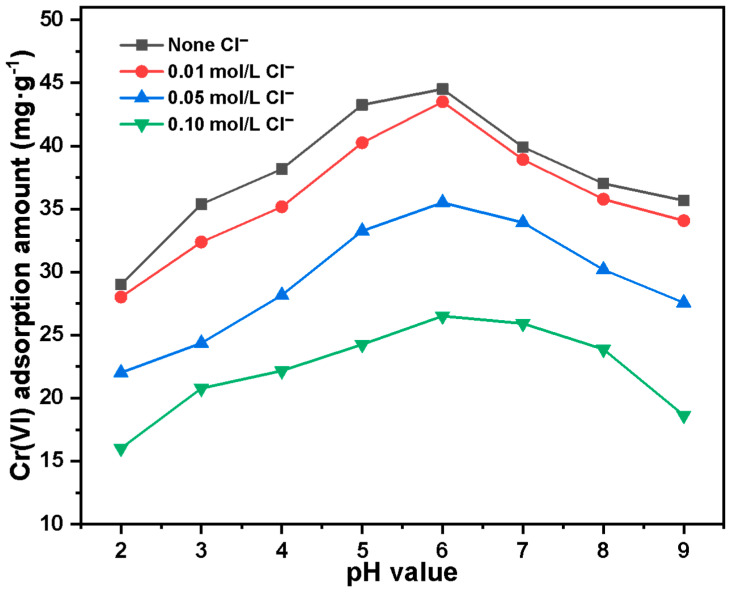
The influence of ionic strength on the adsorption of Cr(VI) by Basalt–MTES/g-C_3_N_4_.

The influence of ionic strength on the adsorption of Cr(VI) by Basalt–MTES/g-C_3_N_4_ is shown in Figure 8. When the Cl^−^ concentration increased from 0 to 0.1 mol·L^−1^, the adsorption amount of Cr(VI) decreased significantly; that is, the increase in the Cl^−^ concentration competed with the Cr(VI) adsorption process, which suggests that the Basalt–MTES/g-C_3_N_4_ adsorption of Cr(VI) may be an anion exchange process.

**Figure 9:**
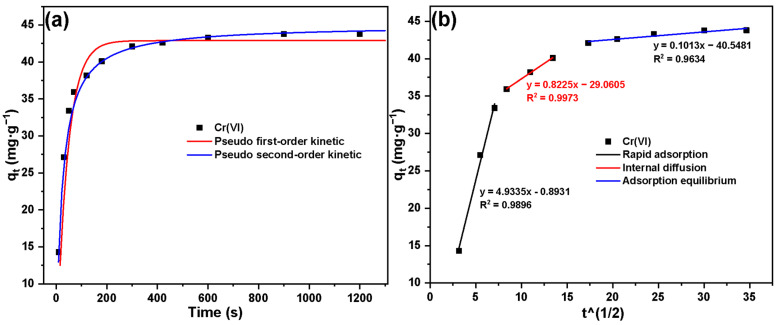
Adsorption kinetic model for Cr(VI) on Basalt–MTES/g-C_3_N_4_ ((**a**): pseudo-first-order and pseudo-second-order kinetic modes; (**b**): intra-particle diffusion model).

The adsorption kinetics results of Basalt–MTES/g-C_3_N_4_ for Cr(VI) are shown in Figure 9. In the variation relationship of ‘adsorption capacity–adsorption time’, the adsorption of Cr(VI) by the material reached equilibrium within 400 s and remained stable in the remaining contact time. It can be seen from the figure that the adsorption rate was relatively fast, within 0 to 90 s. As time went on, a large number of adsorption sites were occupied, resulting in a slowdown in the adsorption rate. After 400 s, the adsorption tended to be balanced.

In order to further explore the adsorption mechanism, three dynamic models (pseudo-first-order kinetic model, pseudo-second-order kinetic model, and intra-particle diffusion) were used; the models were fitted to the curve of Basalt–MTES/g-C_3_N_4_ adsorbing Cr(VI).

The pseudo-first-order kinetic model, pseudo-second-order kinetic model, and intra-particle diffusion model were given as Formulas (1)–(3) [37,38,39]:

(1) $$q_{t}=q_{e}(1-exp(-k_{1}t))$$

(2) $$q_{t}=\frac{q_{e}^{2}k_{2}t}{1+q_{e}k_{2}t}$$

(3) $$q_{t}=k_{p}t^{\frac{1}{2}}+C$$

where q_t_ (mg·g^−1^) is the adsorption capacity at time t (s), and q_e_ (mg·g^−1^) is the equilibrium adsorption capacity. And k_1_ (s^−1^) is the pseudo-first-order kinetic rate constant, and k_2_ (g·s·mg^−1^) is the pseudo-second-order kinetic rate constant. K_p_ is the intra-particle diffusion rate constant.

The fitting results of the pseudo-first-order kinetic model and pseudo-second-order kinetic model are shown in Figure 9a, and the fitting parameters are shown in Table 2. By comparing the correlation coefficient R^2^ and the equilibrium adsorption capacity q_e_, it can be known that the correlation coefficient fitted by the pseudo-second-order kinetic model is higher (R^2^ = 0.9973), and the calculated theoretical adsorption capacity is more consistent with the experimental data, which indicates that the pseudo-second-order kinetic model can better conform to the process of Basalt–MTES/g-C_3_N_4_ adsorbing Cr(VI), reflecting that the rate-determining step of this process might be chemical adsorption [40]. Furthermore, the fitting straight line of the intra-particle diffusion model (Figure 9b) does not pass through the origin, indicating that intra-particle diffusion is not the main factor controlling the adsorption rate. The adsorption of Cr(VI) by Basalt–MTES/g-C_3_N_4_ is mainly based on surface adsorption.

**Figure 10:**
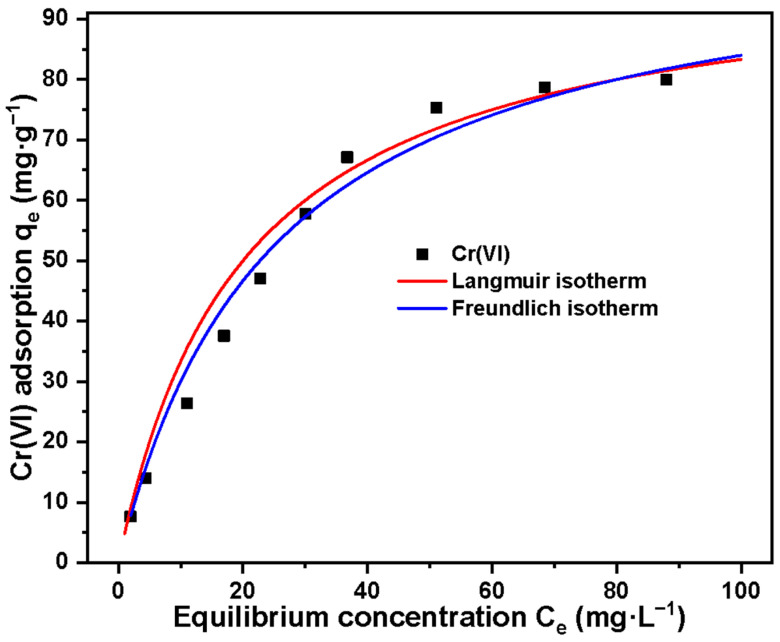
Adsorption isotherm model for Cr(VI) on Basalt–MTES/g-C_3_N_4_.

To determine the interaction between the adsorption material and the adsorbate, we conducted an isotherm study on the adsorption of Cr(VI) by Basalt–MTES/g-C_3_N_4_, as shown in Figure 10. It can be seen from the figure that the adsorption amount of Cr(VI) increases with the increase in the initial concentration of Cr(VI), which is because the higher concentration greatly increases the contact opportunities between the Cr(VI) ions and the active sites on the surface and inside Basalt–MTES/g-C_3_N_4_. The adsorption data were fitted using the Langmuir and Freundlich models, respectively, and the formulas are as follows [41,42]:

(4) $$q_{e}=\frac{q_{max}bC_{e}}{1+bC_{e}}$$

(5) $$q_{e}=K_{f}C_{e}^{n_{f}}$$

where q_e_ (mg·g^−1^) is the equilibrium adsorption capacity, q_max_ (mg·g^−1^) is the maximum adsorption capacity, C_e_ (mg·g^−1^) is the equilibrium concentration, b is the constant, K_f_ is the Freundlich constant, and n_f_ is the concentration index.

The fitting parameters are shown in Table 3 as follows. Through the comparison of the correlation coefficients of the two models, the adsorption process of Cr(VI) by Basalt–MTES/g-C_3_N_4_ is more in line with the Langmuir model. The maximum adsorption amount calculated by the Langmuir model is close to the experimental data. The adsorption behavior conforms to the monolayer adsorption on heterogeneous surfaces, and the interaction between adjacent Cr_2_O_7_^2−^ (or CrO_4_^2−^) can be ignored [43]. The maximum adsorption amount calculated by the Langmuir model was 77.26 mg·g^−1^.

The Van ’t Hoff equation was used to fit the adsorption capacity at different temperatures to obtain the thermodynamic parameters ΔH and ΔS. We calculated the ΔG at different temperatures using the Gibbs equation. The Van ’t Hoff and Gibbs equations were given as Formulas (6) and (7):

(6) $$lnK_{c}=-(\frac{\Delta H}{R})\frac{1}{T}+\frac{\Delta S}{R}$$

(7) ΔG=ΔH−TΔS

and

(8) $$K_{c}=\frac{C_{s}}{C_{e}}$$

where C_s_ is the concentration of the solid surface at the adsorption equilibrium, and C_e_ is the concentration in the solution at the adsorption equilibrium.

**Figure 11:**
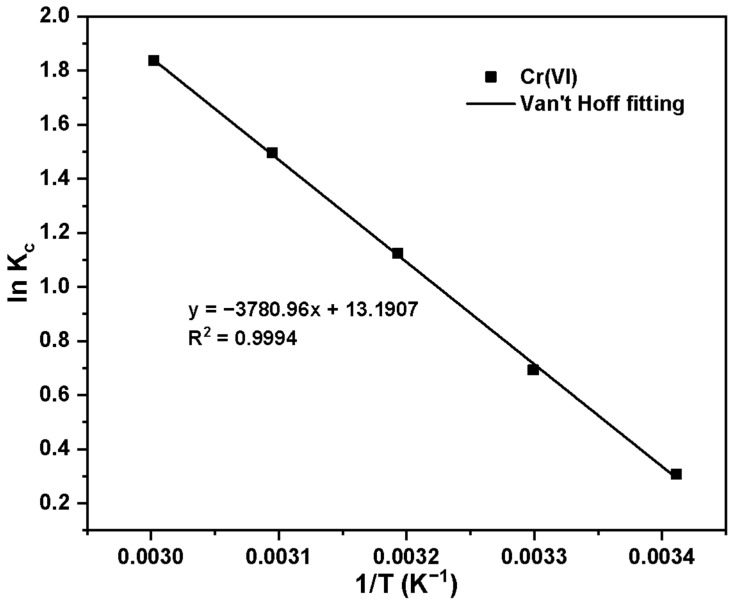
Fitting results of K_c_ and 1/T of Cr(VI).

Van ’t Hoff’s fitting results and parameters for temperature changes are shown in Figure 11 and Table 4. According to the calculation results of the thermodynamic parameters, the ΔH of Basalt–MTES/g-C_3_N_4_ for Cr(VI) adsorption is 31.43 kJ·mol^−1^, which means that the adsorption processes are endothermic. According to the study of Spek, D et al., the enthalpy change range of physical adsorption is between 2.10 and 20.90 kJ·mol^−1^, and the enthalpy change range of chemical adsorption is between 20.90 and 418.40 kJ·mol^−1^ [44]. In the temperature range of 293.15–333.15 K, the ΔG range of Basalt–MTES/g-C_3_N_4_ for Cr(VI) adsorption is between −0.71 and −5.10 kJ·mol^−1^; namely, with the increase in temperature, the ΔG value decreases, indicating that the adsorption process is spontaneous.

### 2.3. Analytical Pretreatment Experimental Result of Cr(VI) by Basalt–MTES/g-C_3_N_4_

**Figure 12:**
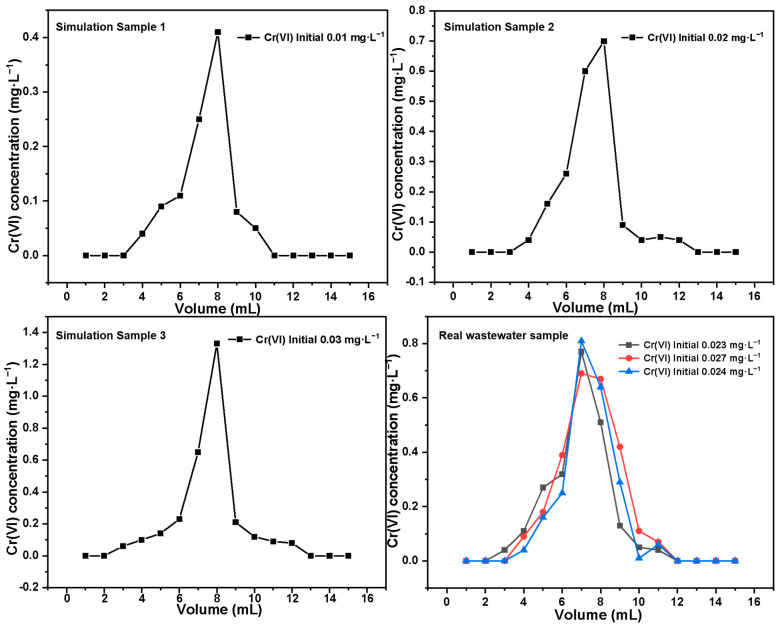
The analytical pretreatment elution curve of Basalt–MTES/g-C_3_N_4_ for Cr(VI).

The analytical pretreatment results (elution curves) of Cr(VI) in simulated samples and real ethylene wastewater samples by Basalt–MTES/g-C_3_N_4_ are shown in Figure 12, and the Cr(VI) recovery rates calculated based on the experiment results are presented in Table 5. According to the calculation results, for the three simulated samples of Cr(VI) with different concentrations (initial concentrations of 0.01 mg·L^−1^, 0.02 mg·L^−1^, and 0.03 mg·L^−1^), the average recovery rates all exceeded 99%. In the tests of the three groups of real wastewater samples, the overall recovery rate was slightly lower than that of the simulated samples, which might be caused by the more complex components in the ethylene wastewater (such as other metal elements and small-molecule organic substances) [45]. However, the recovery rate of Cr in real samples can still reach about 97%. The above experiment results show that for Cr(VI) lower than the detection limit, Basalt–MTES/g-C_3_N_4_ can effectively enrich it by using the extraction chromatographic layer method, thereby achieving the purpose of an accurate analysis.

### 2.4. The Result of the Cyclic Regeneration Experiment

**Figure 13:**
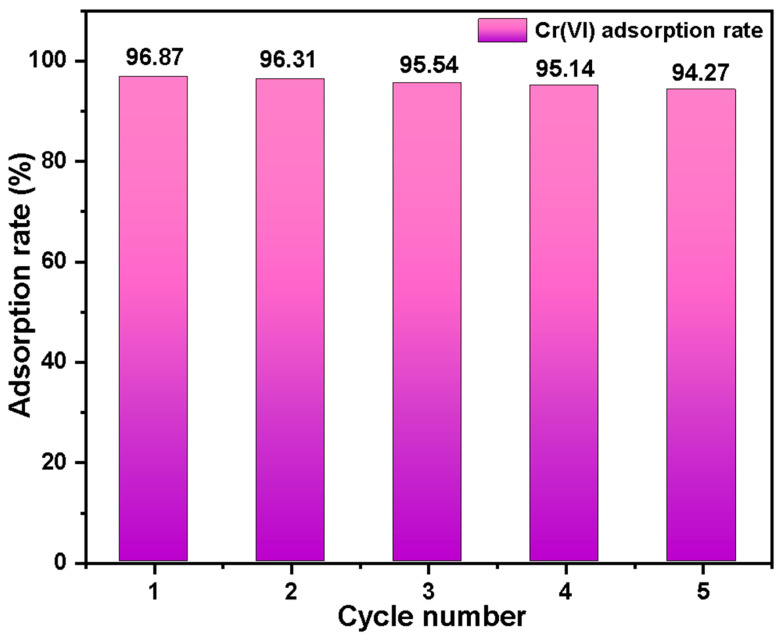
The influence of the number of cycles and regenerations on the removal of Cr(VI) by Basalt–MTES/g-C_3_N_4_.

The Basalt–MTES/g-C_3_N_4_ cycling regeneration experiment is shown in Figure 13. After five cycles, the removal rates of Cr(VI) are 96.87%, 96.31%, 95.64%, 95.14%, and 94.27%, respectively. Under the same experimental conditions, the adsorption capacity of the material slightly decreases with the increase in the number of repeated reactions. This might be due to the fact that the potentially adsorbed Cr(VI) cannot be completely desorbed, resulting in a slight reduction in adsorption sites during the repeated adsorption process. The above results indicate that the Basalt–MTES/g-C_3_N_4_ material has good regeneration ability and reusability and is an effective pretreatment material for Cr(VI) analysis in ethylene wastewater.

## 3. Materials and Methods

### 3.1. Materials

Natural Basalt ore (Chengde Zhongxian Technology Co., Ltd., Longgang, China); melamine (AR, Maclin Reagents Shanghai Co., Ltd., Shanghai, China); hydroxymethyl triethoxysilan (MTES) (98%, Maclin Reagents Shanghai Co., Ltd., Shanghai, China); HNO_3_ (AR, Maclin Reagents Shanghai Co., Ltd., Shanghai, China); anhydrous ethanol (Maclin Reagents Shanghai Co., Ltd., Shanghai, China); K_2_Cr_2_O_7_ solution (1000 mg·L^−1^, Maclin Reagents Shanghai Co., Ltd., Shanghai, China); ethylene cracking untreated wastewater (Sinopec Group, Yanshan Petrochemical Company, Beijing, China).

### 3.2. Preparation of Basalt Matrix-Grafted Graphitic Carbon Nitride Material (Basalt–MTES/g-C_3_N_4_)

#### 3.2.1. Pretreatment of Basalt–MTES

The natural Basalt ore is crushed by a crusher and screened to retain Basalt particles with a size of 80–100 mesh. In the above particles, deionized water is added and then placed in an ultrasonic cleaner for cleaning (20 min) to remove impurities other than the mineral components. After cleaning the Basalt particle material, 2 mol·L^−1^ of HNO_3_ is added, and it is stirred with a magnetic stirrer for 60 min. The above particles are washed with deionized water and dried, and the porous Basalt-based material (Basalt) is prepared for use.

The Basalt was modified with the coupling agent hydroxymethyl triethoxysilane (MTES) to obtain alkylated Basalt. Using xylene as the solvent, the Basalt was placed in a reactor and stirred for 30 min, then MTES was added, and the mixture was stirred and refluxed for 24 h at a constant temperature (80 °C). After the grafting reaction, the product was washed three times with ethanol and dried under vacuum at 60 °C for 24 h to obtain modified Basalt (Basalt–MTES).

#### 3.2.2. Preparation of Basalt–MTES/g-C_3_N_4_

We put 10 g of melamine in an alumina crucible, placed the crucible in a muffle furnace, heated it to 550 °C (2.5 °C/min), and maintained it for 4 h to allow the melamine to react fully. After the reaction was completed, the product was cooled to room temperature in a muffle furnace. We collected the product and grinded it into powder. Then, we rinsed it repeatedly with deionized water and anhydrous ethanol until neutral, and dried it at 70 °C for 24 h. Finally, the dried material was ground and sieved to obtain g-C_3_N_4_.

Using acetic acid solution with a mass fraction of 2.5% as the solvent, Basalt–MTES was put into the reactor and stirred continuously to keep it in a suspended state. Then, we added the g-C_3_N_4_ powder to the above-mentioned mixing system and continued to stir at room temperature for 24 h. Then it was left to stand overnight. After filtration, washing, and freeze-drying, the product was obtained, which was Basalt–MTES/g-C_3_N_4_.

### 3.3. Characterization of Basalt–MTES/g-C_3_N_4_

The morphologies of Basalt, Basalt–MTES, and Basalt–MTES/g-C_3_N_4_ were observed and recorded, respectively, by scanning electron microscopy (SEM, JEM-2100F, Tokyo, Japan); the changes in the surface chemical groups of Basalt, Basalt–MTES, and Basalt–MTES/g-C_3_N_4_ were analyzed by a Fourier Transform Infrared spectrometer (FT-IR, Nicolet-460, Waltham, MA, USA). We mixed Basalt, Basalt–MTES, and Basalt–MTES/g-C_3_N_4,_ respectively, at a ratio of 1:100 evenly, and then placed them on a manual tablet press to make thin slices. The test was conducted under the following conditions: a resolution of 4 cm^−1^, a scanning range of 4000–650 cm^−1^, and 16 scans; the XRD spectra of Basalt, Basalt–MTES, and Basalt–MTES/g-C_3_N_4_ were recorded on a powder diffractometer (D8Quest, Bruker, Zweibrücken, Germany), operating at 40 kV and 40 mA. The elements and chemical states existing in Basalt–MTES/g-C_3_N_4_ were determined by an X-ray photoelectron spectrometer (XPS, Escalalab 250 XI, Thermo Fisher Scientific, Waltham, MA, USA). The specific surface area and pore structure of Basalt and Basalt–MTES/g-C_3_N_4_ were measured on a specific surface area and porosity analyzer (JT-2000, Haixinrui, Beijing, China).

### 3.4. Calculation Method of Adsorption Experiment

The total concentrations of Cr(VI) were determined by a flame atomic absorption spectrophotometer (TAS-990, Puxi, Beijing, China). The adsorption amount Q of Cr(VI) in aqueous solution by the Basalt–MTES/g-C_3_N_4_ was calculated by the following Equation:

(9) $$Q=\frac{\left(C_{0}-C_{e}\right)*V}{m}$$

where Q (mg·g^−1^) is the adsorption amount, C_0_ (mg·L^−1^) is the initial concentration of Cr(VI) before adsorption, and C_e_ (mg·L^−1^) is the concentration of Cr(VI) after adsorption. V (L) is the volume of the solution, and m (g) is the mass of the Basalt–MTES/g-C_3_N_4_.

The removal rate η(%) of Cr(VI) in aqueous solution by the Basalt–MTES/g-C_3_N_4_ was calculated by the following Equation:

(10) $$\eta =\frac{C_{e}}{C_{0}}*100\%$$

### 3.5. Adsorption Experiment of Cr(VI) on Basalt–MTES/g-C_3_N_4_

The influence of Basalt–MTES/g-C_3_N_4_ on the removal of Cr(VI) under different factors was investigated by a single-factor analysis method to determine the adsorption performance of the material. These experiments mainly include pH and ionic strength, adsorption kinetics research, adsorption isotherm research, and adsorption heat research. After each group of adsorption experiments was completed, the adsorbent was separated by filtration, and the supernatant was taken to determine the concentration of Cr(VI). The adsorption capacity Q_e_ and the removal rate η of Cr(VI) were calculated using Formulas (9) and (10).

### 3.6. Analytical Pretreatment Experiment of Cr(VI)

A certain amount of Basalt–MTES/g-C_3_N_4_ was taken, soaked in deionized water for 24 h, and then filtered by suction. Then, it was washed multiple times with 1 mol·L^−1^ HNO_3_ until the washing liquid was colorless and transparent. We washed with deionized water until neutral, and vacuum dried at 80 °C until constant weight was ready for use. The pretreated Basalt–MTES/g-C_3_N_4_ was loaded into a 5 mL extraction chromatographic column, and the column was passed through with HNO_3_ (pH = 5.0) to pre-balance it and set it aside for later use. The natural flow rate of the chromatographic column is approximately 0.5–0.6 mL·min^−1^. Note: The analytical measurement instrument used in this experiment is a flame atomic absorption spectrophotometer (TAS-990, Puxi, Beijing, China). Experimental verification shows that the detection limit of Cr measurement by this instrument is 0.037 mg·L^−1^.

Simulation sample experiment: We prepared 100 mL of Cr(VI) solutions with concentrations of 0.01 mg·L^−1^, 0.02 mg·L^−1^, and 0.03 mg·L^−1^, respectively (all concentrations were less than the detection limit of Cr by the instrument) and pH = 5.0. We passed each of the above solutions through the Basalt–MTES/g-C_3_N_4_ extraction chromatographic column, respectively, then eluted Cr(VI) with a 0.1 mol·L^−1^ Na_2_C_2_O_4_ solution, and collected 15 mL of the eluent (collected 15 times, 1 mL each time) [14]. The eluent concentrations of each group were measured, respectively, by a flame atomic absorption spectrophotometer, as mentioned above. Each group of experiments was repeated three times, and the elution curve of Cr(VI) was plotted.

Real wastewater sample experiment: We randomly took three oily water samples from the ground produced by ethylene cracking (with a pH of approximately 5.0). It was known, through concentration analysis, that the initial Cr concentrations were 0.023 mg·L^−1^, 0.027 mg·L^−1^, and 0.024 mg·L^−1^, respectively. In total, 100 mL of a water sample was passed through the Basalt–MTES/g-C_3_N_4_ extraction chromatographic column, and then Cr(VI) was eluted with a 0.1 mol·L^−1^ Na_2_C_2_O_4_ solution [14]. We collected 15 mL of the eluent (collect 15 times, 1 mL each time). We measured the concentration of the eluent and plotted the elution curve of Cr(VI).

### 3.7. Recycling and Regeneration Experiment

The Basalt–MTES/g-C_3_N_4,_ after adsorbing Cr(VI), was added to a conical flask containing the Na_2_C_2_O_4_ solution (0.1 mol·L^−1^) and desorbed through constant-temperature oscillation for 12 h to dissociate Cr from the surface or interior of the adsorbent. After desorption, we took a certain volume of the supernatant, measured the concentration of Cr(VI), and calculated its removal rate of Cr(VI). The desorbed material was washed with deionized water until neutral, dried, and then repeatedly adsorbed five times under the same conditions.

## 4. Conclusions

This study successfully prepared a porous Basalt–MTES/g-C_3_N_4_ composite by grafting g-C_3_N_4_ onto acid-purified natural Basalt via MTES coupling agents for efficient Cr(VI) enrichment in ethylene wastewater. Characterization revealed uniformly distributed g-C_3_N_4_ nanoparticles on the Basalt surface, with 82.63% melamine participating in crystallization, while the material exhibited a high specific surface area (403.55 m^2^/g) and mesoporous structure (34.29 nm). Adsorption experiments demonstrated that protonated amino groups in g-C_3_N_4_ under acidic conditions (pH 5–6) dominated Cr(VI) removal through anion exchange. The process followed pseudo-second-order kinetics and Langmuir monolayer adsorption mechanisms. The composite achieved >97% recovery of trace Cr(VI) via chromatographic extraction and maintained 96.87% removal efficiency after five regeneration cycles. This work proposes a cost-effective, recyclable green pretreatment strategy for high-sensitivity Cr(VI) detection in ethylene industrial wastewater, offering dual benefits in environmental remediation and industrial compliance. The design integrates Basalt’s structural stability with g-C_3_N_4_’s adsorption functionality, advancing sustainable wastewater treatment technologies.

## Figures and Tables

**Table 1:** Pore size and specific surface area before and after the grafting reaction.

Species	Pore Size (nm)	Specific Surface Area (m^2^·g^−1^)
Basalt	71.27	563.25
Basalt–MTES/g-C_3_N_4_	34.29	406.55

**Table 2:** Adsorption kinetic parameters (pseudo-first order and pseudo-second order) for Cr(VI) on Basalt–MTES/g-C_3_N_4_.

Adsorption Kinetic Model	Parameters	Cr(VI)
Pseudo-first order	q_e_ (mg·g^−1^)	55.28
	K_1_ (s^−1^)	0.014
	R^2^	0.985
Pseudo-second order	q_e_ (mg·g^−1^)	42.62
	K_2_ (g·s·mg^−1^)	0.086
	R^2^	0.997

**Table 3:** Langmuir and Freundlich adsorption isotherm parameters for Cr(VI) on Basalt–MTES/g-C_3_N_4_.

Adsorption Isotherm Model	Parameters	Cr(VI)
	q_max_ (mg·g^−1^)	77.26
Langmuir	b (L·mg^−1^)	0.093
	R^2^	0.992
Freundlich	K_f_	8.538
	n_f_	0.354
	R^2^	0.946

**Table 4:** Adsorption thermodynamic parameters of Cr(VI).

Species	T (K)	ΔG (KJ·mol^−1^)	ΔH (KJ·mol^−1^)	ΔS (J·mol^−1^·K^−1^)	R^2^
Cr(VI)	293.15	−0.71	31.43	109.67	0.9994
	303.15	−1.81			
	313.15	−2.91			
	323.15	−4.02			
	333.15	−5.10			

**Table 5:** Statistics on the recovery rate of Cr(VI) in simulated samples and real samples.

Species	Initial Cr(VI) Concentration of the Sample		Enrichment Rate
Cr(VI)	Simulated sample	0.01 mg·L^−1^	99.21%
		0.02 mg·L^−1^	99.54%
		0.03 mg·L^−1^	99.33%
	Real sample	0.023 mg·L^−1^	97.31%
		0.027 mg·L^−1^	98.02%
		0.024 mg·L^−1^	97.24%

## Data Availability

Please visit the website for relevant data: https://app.globus.org/file-manager?origin_id=82f1b5c6-6e9b-11e5-ba47-22000b92c6ec&origin_path=%2Ftmp%2F (accessed on 1 June 2025). After entering, click to select: Data—Grafting Graphite-phase Carbon Nitride Material based on Coupling Agent Hydroxymethyl triethoxysilane Modified Basalt Matrix.
